# Socio-economic status over the life course and obesity: Systematic review and meta-analysis

**DOI:** 10.1371/journal.pone.0177151

**Published:** 2017-05-16

**Authors:** Suzy Newton, Dejana Braithwaite, Tomi F. Akinyemiju

**Affiliations:** 1 Department of Epidemiology, University of Alabama at Birmingham, Birmingham, Alabama, United States of America; 2 Division of Cancer Epidemiology, Department of Epidemiology and Biostatistics, University of California San Francisco, San Francisco, California, United States of America; 3 Comprehensive Cancer Center, University of Alabama at Birmingham, Birmingham, Alabama, United States of America; University of Texas Southwestern Medical Center at Dallas, UNITED STATES

## Abstract

**Background:**

The purpose of this review was to summarize the published literature on the association of childhood, adulthood and life course socio-economic status (SES) with obesity between January 1990 and June 2015.

**Methods:**

The major medical electronic databases were searched to identify studies that examined SES over the life-course in relation to obesity. A total of 219 studies were identified through the initial search, and 35 qualified for full review. Of these, 14 publications met our inclusion criteria for the meta-analysis, all from developed or upper-middle income countries.

**Results:**

There was a consistent association between lower life course SES and obesity among women (summary OR: 1.35, 95% CI: 1.04, 1.76), but not among men (summary OR: 0.92, 95% CI: 0.60, 1.40). Overall, mean BMI was higher among individuals with lower life course SES compared with those with higher life course SES (summary mean BMI difference: 0.65, 95% CI: 0.59, 0.71). Mean waist circumference (WC) was higher among women with lower life course SES compared with those with higher life course SES (summary mean WC: 4.67, 95% CI: 4.15, 5.20), but lower among men (summary mean WC difference: -0.10, 95% CI: -0.11, -0.08).

**Conclusion:**

The inverse relationship between life course SES and obesity among women was consistent, based mostly on studies in developed countries. Nevertheless, critical information gaps remain in relation to the impact of childhood and life course SES on obesity in developing countries.

## Background

Although the global prevalence of obesity nearly doubled between 1980 and 2008, obesity rates have risen faster in low- and middle- income countries (LMICs) compared with high income countries [1]. Recent transitions in nutrition and lifestyle in many LMICs have led to increased life expectancy, but also to increased consumption of high-fat and high-calorie diets and physical inactivity, mirroring trends observed in high-income countries several decades ago[2]. Renewed attention has been paid to the importance of social inequalities in health, and a recent Institute of Medicine report recognized that chronic disease risk factors, such as obesity, are likely shaped over the life course during critical windows of development from childhood to adolescence [3]. Scientific evidence suggests that social class affects health [4–9], and cumulative stress beginning in infancy predicts risk factors, leading to development of chronic diseases [10]. These observations, coupled with evidence of increasing global prevalence of obesity and obesity-associated chronic diseases, which are also socio-economically patterned, highlight the importance of understanding the socio-economic patterns of obesity over the life course.

Current recommendations from the World Cancer Research Fund (WCRF) state that median adult body mass index (BMI) be maintained between 21-23kg/m^2^, based on population-specific normal ranges [11]. As countries transition from low to middle income, understanding the demographic and socio-economic patterns of obesity across the life course will be critical for avoiding the obesity trend observed as middle-income countries transition to high-income[2]. Progress on reducing obesity and obesity-associated chronic diseases requires a thorough understanding of risk factors starting in early life in order to inform obesity prevention strategies that have the best chance of success. In this systematic review and meta-analysis, we summarize the existing literature on the link between life course SES and obesity, and assessed similarities and differences between studies from developed and developing countries.

## Methods

### Data sources and literature search

We conducted a systematic review of previously published studies between January 1990-June 2015 through the PUBMED, MEDLINE, EMBASE and CINAHL databases, following established PRISMA guidelines. We used relevant text words and medical subject heading (MESH) terms to identify relevant studies, including all iterations of ‘Life course’, ‘socioeconomic’, ‘social class’, ‘social accumulation’, ‘father’s’, ‘mother’s’ combined with any of the following keywords: ‘body mass index’, ‘obesity’, ‘adiposity’, and ‘weight change’. Included studies were those focused on adults and published in the English language. Data for this study was obtained from published articles, and was therefore exempt from ethical review.

### Study selection and data extraction

A flowchart depicting the process of study selection according to PRISMA guidelines is detailed in S1 Fig. Two authors (SN and TA) independently reviewed study titles, abstracts and full text, and discrepancies in selected articles were resolved following a discussion by both authors. Studies were included in the analysis if they reported a quantitative estimate (e.g. odds ratio (OR), mean BMI, prevalence) and standard errors (SE or 95% confidence interval) between life course SES and a measure of obesity (weight, BMI, waist-to-hip ratio, adiposity). If there was no data on confidence interval or standard error of an estimate presented, the sample size of each group had to be present to be included in our analysis. Studies were excluded if they met at least one of the following criteria: (1) no relevant data presented; (2) non-English language; (3) other outcomes; (4) not original research; (5) no life course SES; or (6) published outside the range of 1990-June 2015. Data from each selected study was abstracted into an electronic database and independently verified against the original articles. Study data extracted included: author name and year of publication, country of the study, study design, study population demographics, sample size, SES construct, BMI or obesity measure and study covariates. We extracted estimates and 95% CI or standard errors for the two most extreme categories of life course SES- comparing the highest SES categories over the life course with the lowest.

### Statistical analysis

We calculated the mean BMI, mean waist circumference (WC) and odds ratios (ORs) for BMI comparing the lowest life course SES category with the highest life course SES category for each study. When relative estimates were reported comparing the opposite contrast i.e. highest SES compared with lowest, we calculated the inverse to correspond to lowest SES vs. highest SES. We estimated summary rate ratios and mean BMI estimates comparing low life course SES with high life course SES using random-effects models. We assessed heterogeneity across studies using the Cochran Q-statistic, and used the I^2^ statistic to approximate the proportion of total variation in the estimates due to between-study heterogeneity. We conducted subgroup analysis by gender, (males, females, all genders), and tests of heterogeneity between subgroups were estimated using meta-regression analysis. Potential publication bias was assessed via visual inspection of funnel plots as well as with the Egger’s test. We present here the results of the Egger’s test. All statistical analyses were performed using STATA software, version 12.0 (Stata Corp, College Station, Texas USA).

## Results

### Literature search and included studies

**Fig 1:**
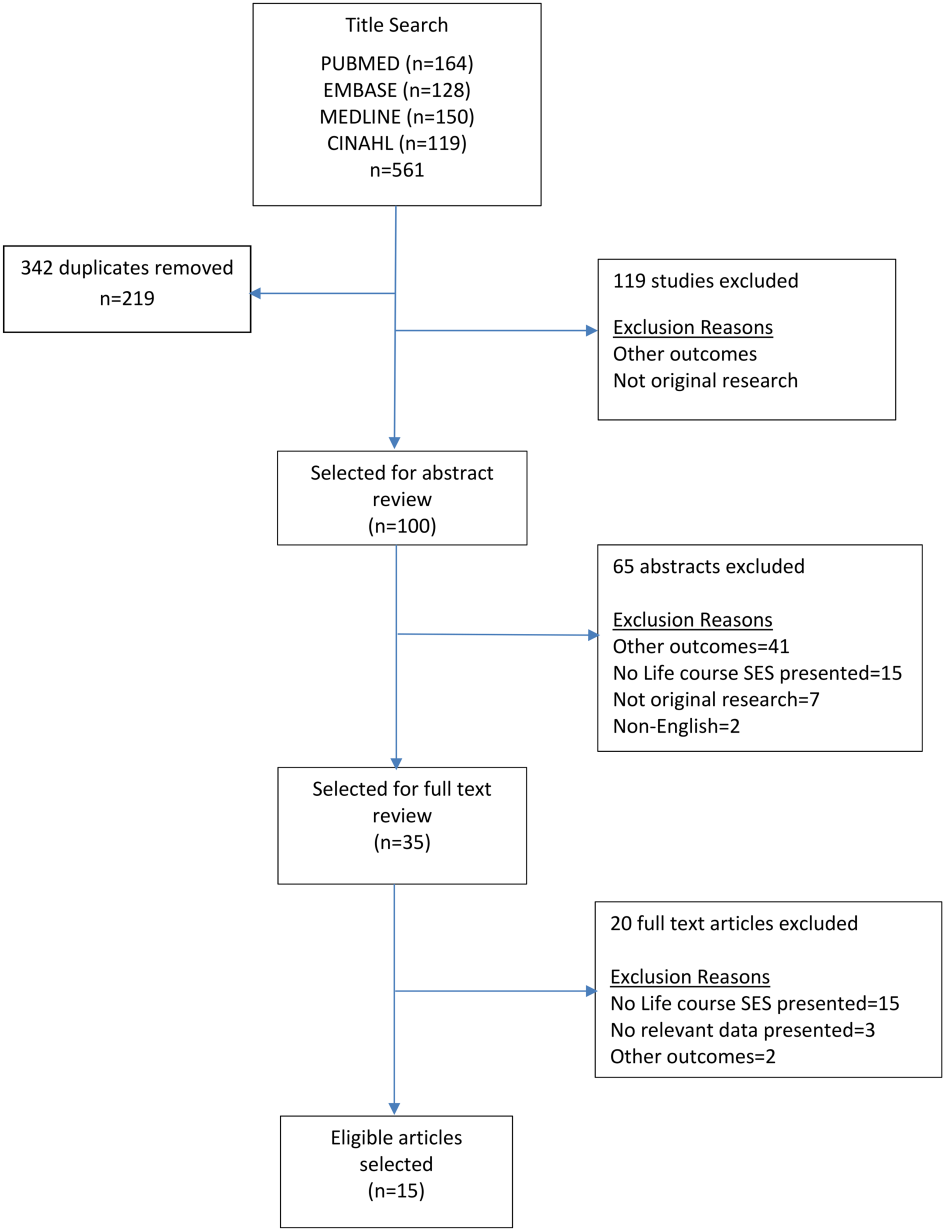
Publication search and selection results.

We identified 219 unique studies through our literature search, and reviewed 35 studies in full (Fig 1). After full-text review, 15 studies were eligible for inclusion in Table 1[4, 12–25]. One study did not report data required for calculating confidence intervals, and therefore was not included in the calculation of summary estimates using meta-analysis but was included in the systematic review since it met the study inclusion criteria[25]. Most of the studies were published between 2003 and 2014[4, 12–24]. Three out of the 15 were based on US populations [19, 20, 22], while others included study populations from the UK[13, 17, 18, 23], Denmark[15], Brazil[12, 21], Singapore[16], Scotland[14, 25], Australia[4], and Spain[24]. Seven studies were longitudinal in design [4, 12, 13, 15, 17, 19, 21], and eight were cross-sectional [14, 16, 18, 20, 22–25]. Life course SES was evaluated using self-reported data in only two studies [4, 15], while the majority used a combination of self-reported and primary data [12–14, 16–20, 22–25]. Data source was not reported in one study [21]. Eight studies reported life course SES based on father’s (or the primary caregiver’s) occupation [13, 14, 17, 18, 22–25], others reported life course SES based on childhood family income [12, 16, 21], while others used a combination of parental occupation, family income and/or parental education [4, 15, 19, 20]. One study [25] obtained data from males only, three studies [4, 22, 23] on females only, while 11 studies [12–21, 24] obtained data from both male and female participants. Nine studies [14–16, 18–20, 22, 23, 25] provided estimates of the association between life course SES and measures of obesity that were adjusted for confounders, while six studies [4, 12, 13, 17, 21, 24] reported only unadjusted estimates.

**Table 1:** Studies reviewing life course SES and obesity, published 1990–2015.

Author, year	Country	Design	Population	Measure of Lifecourse SES	N	Estimate Low vs. High SES	Covariates	Conclusion
Boylan, 2014[15]	Denmark	Longitudinal	Male and female teenagers born 1964–1969	Father's occupation, father's education+own occupation, own education	786	OR (CI): M: 1(.3–2.9); F: 2.8(.9–8.3)	adult energy intake, physical activity, smoking, adolescent/parental BMI, adult/adolescent SES	Women in the stable low lifecourse SES group were more likely to be obese compared with those in the stable high SES. Men's BMI remain unchanged between the groups.
Aitsi-Selmi, 2013[12]	Brazil	Longitudinal	Males and females recruited at birth in 1978/79	Childhood family income+current family income	2063	Mean BMI (SD): M: 24.8(4.6) vs. 25.4(4.7); F: 24.6(5.8) vs. 22.6(4.1); Mean WC (SD): M: 86.8(12.0) vs. 88.9(12.4); F: 79.4(13.1) vs. 73.9(9.7); Mean Waist-Hip Ratio (SE): M: .86(.06) vs .86(.06); F:.78(.06) vs. .74(.06)	None	Men in the persistently low lifecourse SES group had a slightly lower BMI on average than men in the persistently high lifecourse SES group, however women in the persisently low lifecourse SES group had a higher BMI than those in the high group.
Malhotra, 2013[16]	Singapore	Cross-sectional	Males and females aged 60+	Family financial status+own education	3566	OR (CI): M: .68(.38–1.22); F: 1.32(.88–1.99)	age	Men were less likely to be obese if they were in low lifecourse SES, while results for women were opposite but non-significant.
Murray, 2011[17]	UK	Longitudinal	Males and females recruited at birth, 1946	Father's occupation+own occupation	3035	Mean BMI (SE): M: 27.9(4.2) vs. 26.7(3.8); F: 28.6(6.3) vs. 26(4.4)	None	Men and women in the persistent low lifecourse SES groups had higher BMI than those in the persistent high lifecourse SES groups
Heraclides, 2010[18]	England	Cross-sectional	Males and females aged 44–69	Father's occupation+own education, own occupation	4598	OR(CI): M: 1.25 (1.0–1.55)); F: 2.61 (1.79–3.78)	age	Women and men with low lifecourse SES were more likely to be obese compared with those of high lifecourse SES.
Scharoun-Lee, 2009[19]	US	Longitudinal	M and F adolescents in grades 7–12	Parental material endowments, skills, knowledge, material, human, and social capital+own of above	12940	RRR obesity (BMI) incidence (CI): M: 1.18(.82–1.7); F: 3.01(1.95–4.66); RRR obesity (BMI) persistence (CI): M: 1.98(1.25–3.15); F: 3.56(2.01–6.3)	age	Men were slightly more likely to be obese, and women were 3 times more likely to be obese if they were in the persistent low lifecourse SES compared to high lifecourse SES.
Hart, 2008[14]	Scotland	Cross-sectional	Males and females aged 30–59	Parental occupation+own occupation	2338	PR(CI): M: 1.28(1.09–1.51); F: 1.92(1.63–2.26); Mean WC (SE): M: 93.3(11.9) vs. 93.4(10); F: 83(13.2) vs. 78.9(12.3)	age	Men and women in the stable low SES group had higher BMI than those in the stable high SES group
Bennett, 2007[20]	US	Cross-sectional	African American males and females aged 25–50 at baseline	Parental occupation, material household conditions+own education, occupation, employment status, homeowner	1178	Mean BMI at baseline (SE): M: 25.7 (0.3) vs. 26.6(.8); F: 30.0(.4) vs. 27.3(1); Mean BMI at followup (SE): M: 28.7(.4) vs. 30.6 (1.0); F: 34.5(.5) vs. 33.9(1.3); Mean BMI change (SE): M: 3.1(.3) vs. 4.0(.7); F: 4.5(.3) vs. 6.6(.9)	age	Men who were in the low lifecourse SES group had lower BMI, however women in the low lifecourse SES group had higher BMI at baseline compared with those in the high lifecourse SES.
Ball, 2006[4]	Australia	Longitudinal	Females aged 18–23 years at baseline	Father's education+own education; Mother's education+own education; Father's occupation+own occupation; Mother's occupation+own occupation	8756	Father edu. Mean BMI (SD): 24.3(5.2) vs. 22.8 (4.2); Δweight (SE): 2.9(7.3) vs 2.0(5.9); Mother edu. Mean BMI (SD): 24.2 (5.2) vs 22.9(3.9); Δweight (SE): 2.9(7.3) vs 1.9(5.7); Father occ. Mean BMI (SD): 24.2(5.1) vs. 23(4.2); Δweight (SE): 2.6(6.9) vs 2.2(6.0); Mother occ. Mean BMI(SD): 24(5) vs. 23 (4.2); Δweight (SE): 2.5(6.9) vs 2.0(5.9)	none	Average BMI was higher among women in the low lifecourse SES groups compared to higher lifecourse SES groups based on both mother and father education or employment.
Barros, 2006[21]	Brazil	Longitudinal	Males and females recruited at birth, 1982	Childhood family income+current family income	1031	PR (CI): M: .42(.36-.49); F: 1.4(1.2–1.63)	none	The prevalence of overweight was higher among women who were always poor compared with those who were never poor. The opposite was true for men.
James, 2006[22]	US	Cross-sectional	African American females aged 25–50	Parental occupation+own education, occupation, employment status, housing status	679	OR (CI): 2.12(.75–6.0)	age, marital status, alcohol, smoking, childhood food insecurity, fruit/veg consumption, strenuous exercise	Women in the stable low lifecourse SES group had twice the odds of obesity compared to women in the stable high lifecourse SES group, but this was not statistically significant
Ebrahim, 2004[23]	UK	Cross-sectional	Females aged 60–79 years	Father's occupation+ own occupation	2936	PR (CI): 1.88(1.63–2.87)	age	The prevalence of obesity was higher among women with low lifecourse SES (adult and childhood manual occupation) compared with high lifecourse SES.
Regidor, 2004[24]	Spain	Cross-sectional	Males and females aged 60 and older	Father's occupation+own occupation	4009	General obesity PR (CI): M: 1.01 (.84–1.21); F: 1.21(1.06–1.38); Abdominal obesity PR (CI): M: 1.02(.9–1.16); F: 1.11(1.04–1.18)	none	The prevalence of general obesity was higher among women of low lifecourse SES (working class childhood and adulthood) compared with high lifecourse SES, but not among men
Langenberg, 2003[13]	UK	Longitudinal	Males and females recruited at birth in 1946	Father's occupation+own occupation	3035	Mean BMI (SE): M: 27.7(.20) vs. 26.8(.18); F: 28.8(.28) vs. 26.1(.26); Mean Waist Hip Ratio (SE): M: 94.9(.31 vs. 92.5(.48); F: 81.9(.31) vs. 79.6(.31); Mean WC (SE): M: 98.5(.54) vs. 96.6(.51); F: 89(.66) vs. 83.1(.59)	none	Mean BMI was higher among men and women with low lifecourse SES (manual father and adult social class) compared with those with high lifecourse SES
Blane, 1996[25]	Scotland	Cross-sectional	Males aged 35–64	Father's occupation+own occupation	5645	Mean BMI: 25.3 vs. 24.9	age	Men who remained in a low lifecourse SES had higher BMI compared to men who remained in a high lifecourse SES

BMI, body mass index; M, males; F, females; WC, waist circumference; OR, odds ratio; CI, confidence interval; SE, standard error; RRR, relative risk ratio; PR, prevalence ratio; edu, education; occ, occupation

### Association between life-course SES and mean BMI

**Fig 2:**
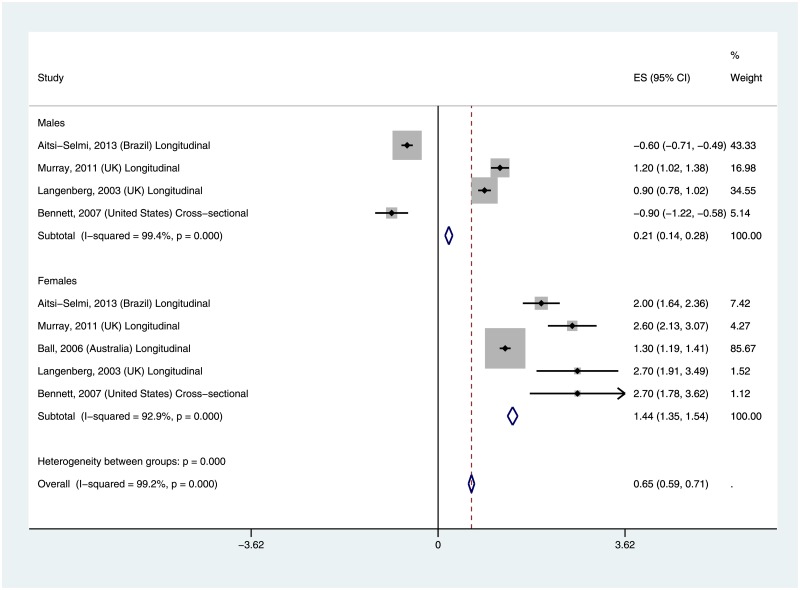
Mean BMI difference by life course SES.

Five studies reported an association between life course SES and mean BMI[4, 12, 13, 17, 20], four included data from both males and females [12, 13, 17, 20], and one study included only females [4]. Four of the studies were conducted in the developed countries of US, UK and Australia [4, 13, 17, 20], while one study was conducted in Brazil [12]. The results of the meta-analysis examining the association between life course SES and adult BMI are displayed in Fig 2. Males with lower life course SES had slightly higher mean BMI compared with those of higher life course SES; the pooled estimate of the mean BMI difference was 0.21 (95% CI: 0.14–0.18). Among females, there was a larger mean BMI difference among those of lower life course SES compared with those of higher life course SES; the pooled estimate of the mean BMI difference was 1.44 (95% CI: 1.35–1.54). Overall when both genders were combined, mean BMI remained higher among participants with lower life course SES compared with those of higher life course SES: 0.65 (95% CI: 0.59–0.71). Results for females were consistent across all included studies, while two studies for males reported lower mean BMI among lower life course SES adults (63, 44). One of the two studies was a longitudinal study from Brazil[12] while the other was a cross-sectional study from the U.S.[20]. There was evidence of significant heterogeneity across the included studies (I^2^ = 99%, p-value = 0.00) overall, and for both males (I^2^ = 99%, p-value = 0.00) and females (I^2^ = 94%, p-value = 0.00). Sensitivity analysis excluding the only study with a cross-sectional design had minimal effects on the finding.

### Association between life course SES and BMI categories

**Fig 3:**
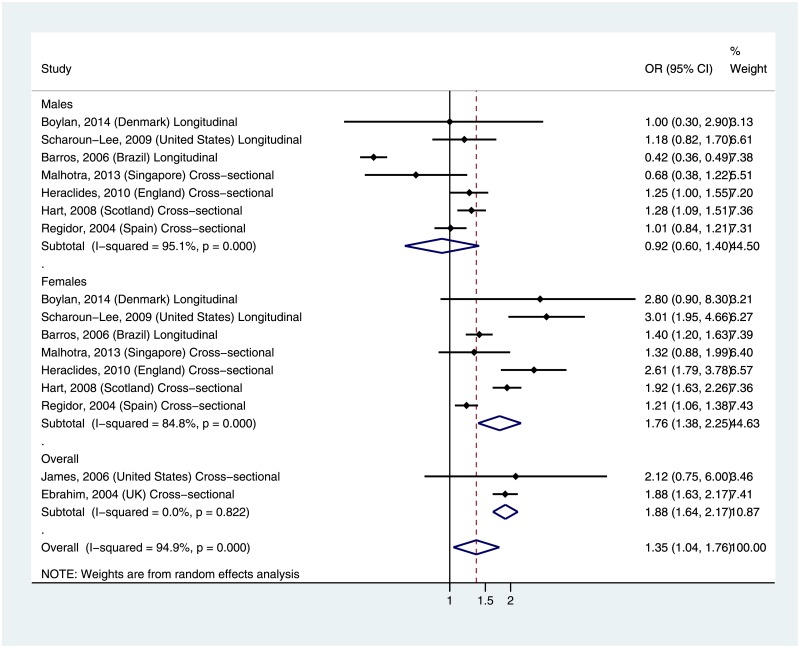
Summary odds ratio for obesity by life course SES.

Seven studies examined life course SES in relation to the odds of obesity (Fig 3) among males and females [14–16, 18, 19, 21, 24], while two other studies examined the same association but results were not stratified by gender [22, 23]. The studies represented in the analyses were mostly from developed countries, including Denmark, US, Scotland, Spain, England, UK, Singapore, and one middle-income country- Brazil. There was no significant difference in the odds of obesity by life course SES among males (Summary OR: 0.92, 95% CI: 0.60–1.40). In contrast, females of lower life course SES had significantly higher odds of obesity compared with those of higher life course SES (Summary OR: 1.76, 95% CI: 1.38–2.25). Two studies reported estimates that were un-stratified by gender [22, 23], and also showed a higher summary odds ratio of obesity among lower life course SES compared with higher life course SES (Summary OR: 1.88, 95% CI: 1.6402.17). The overall summary OR across all included studies was 1.35 (1.04–1.76). While all of the included studies for females reported higher OR for obesity among lower life course SES participants, two out of the seven studies of males reported lower ORs among lower life course SES participants; a longitudinal study conducted in Brazil [21] and a cross-sectional study from Singapore [16]. There was evidence of significant heterogeneity across all of the included studies (I^2^ = 95%, p-value = 0.00) and for both males (I^2^ = 95%, p-value = 0.00) and females (I^2^ = 85%, p-value = 0.00). However, there was no evidence of heterogeneity in the two studies that were un-stratified by gender (I^2^ = 0.0%, p = 0.82). Both studies were cross-sectional designs, and based in the U.S. and U.K.

### Association between life course SES and waist circumference

**Fig 4:**
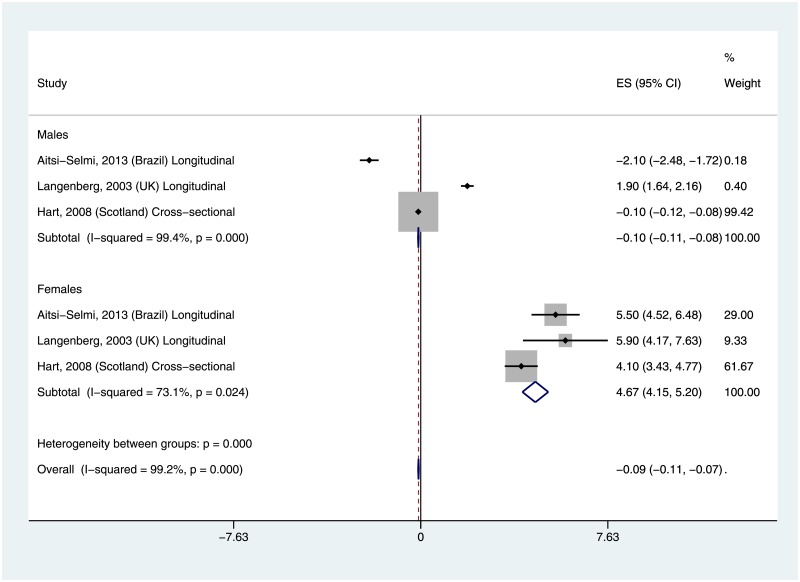
Mean difference in waist circumference by life course SES.

Three studies examined life course SES in relation to waist circumference (WC) among males and females [12–14]. The three studies were conducted in Brazil, UK and Scotland. Two [12, 14] of the three studies showed lower mean WC among lower life course SES males, while the other study [13] showed higher mean WC among lower life course SES males (Fig 4). The summary mean WC comparing lower life course SES with higher life course SES among males was -0.10 (95% CI: -0.11, -0.08). All three studies showed significantly higher WC among lower life course SES compared with higher life course SES females, with summary mean WC of 4.67 (95% CI: 4.15, 5.20). Overall, the summary WC comparing lower life course SES adults with higher life course SES adults was -0.09 (-0.11, -0.07). There was evidence of significant heterogeneity across all of the included studies (I^2^ = 99%, p-value = 0.00) and for both males (I^2^ = 99%, p-value = 0.00) and females (I^2^ = 73%, p-value = 0.02).

## Discussion

We focused our review on the influence of SES across the life course on obesity. Whereas we observed that there were somewhat consistent findings for the association between life course SES and obesity in developed countries[4, 13, 14, 16, 18–20, 22, 23, 26, 27], there was very little information available for developing countries. We conducted a systematic review and meta-analysis to synthesize the current evidence in this area and identify gaps in the literature, focusing on studies that directly examined SES change over the life course. The results of our meta-analysis were highly consistent with previous findings. Women with higher SES throughout their life have lower BMI, while findings among men were less consistent. One possible reason for the differing association is that females may have weight-related ideals that are easier to maintain with higher income[28], and that these ideals may not exist for men[29]. While another possible reason for this difference is the fact that low SES men might engage in higher levels of physical activity because they engage in more manual occupational labor[30], this does not take into account the effect of childhood SES.

Most of the studies were conducted in developed countries (13 out of 15), with the exception of two studies that were conducted in Brazil[12, 21], an upper-middle income country according to the World Bank Income Classification. Therefore, it was not surprising that findings from the Brazilian studies were similar to results of other developed countries, i.e. women were more likely to be obese if they had lower life course SES. This is consistent with Monteiro’s and Dinsa’s reviews indicating that the association between SES and obesity becomes inverted as countries transition into higher income [31, 32]. Singapore was an interesting inclusion in the meta-analysis, because the majority of the participants, all aged ≥ 60 years, would have spent all or most of their childhood during the period 1912–1965, during which time Singapore was considered to be a developing country [16]. While the study from Singapore revealed that men were more likely to be obese if they had higher life course SES, women were more likely to be obese if they had lower life course SES, similar to patterns observed in developed countries. Further research is needed to illuminate the socio-economic transition that occurred in Singapore, and the impact of obesity and associated chronic diseases in adulthood.

The studies included in this review sought to determine the influence of SES on obesity from childhood through adulthood, providing a comprehensive investigation of reported differences in both developed and developing countries. Although some studies find that the effects of childhood SES on adult obesity were attenuated when adult SES was accounted for[4, 6, 13, 14, 20, 22, 23, 27, 33–45], other studies nevertheless highlight the importance of considering the lasting effects of childhood social class, notably Tucker-Seeley et al.’s 2011 study (74) on multi-morbidity among older adults. In this study, participants who experienced childhood financial hardship were slightly more likely to suffer from chronic conditions in adulthood compared with those who did not report childhood financial hardship[46]. Another recent study linked childhood SES to breast cancer incidence and mortality[47], and a systematic review on the association between childhood SES and cause-specific mortality concluded that mortality risk for all causes was higher among those who experienced poor SES during childhood[48]. In the current review, the vast majority of studies have been in developed countries, with a few studies in middle-income countries where the association between childhood SES and adult obesity remains inconsistent. This highlights the need for more studies in developing countries, where chronic disease rates have risen significantly and are expected to outstrip infectious diseases as the major cause of death in a few decades. In all regions, further studies are needed to develop public health strategies aimed at mitigating the impact of low childhood SES on adult health.

Several publications from countries in both developed and developing countries suggest that there may be opposite, but consistent associations between adult SES and obesity which may also vary by gender. Several reviews of the literature have been conducted focusing on adult SES and obesity across countries, with most concluding that there are no consistent associations between obesity and SES among men [8, 31, 32]. High-income countries consistently show an inverse relationship between adult SES and obesity[4–9, 49]. Data from middle-income countries are somewhat less consistent, but this is most likely explained by the views of Monteiro (27) and Dinsa (31) in previous literature reviews, in which the authors concluded that the association between SES and obesity in women changes from a positive association to an inverse association as a country’s GDP increases [31, 32]. This view is supported by other studies[8, 50–52] that include LMICs. Older studies have characterized a positive, i.e. direct, association between BMI and SES[9, 53–55], while more recent studies suggest this link is complicated and changes as nations become more developed. The decreasing levels of obesity in higher SES women observed in more recent studies could be attributed to the availability of more variety in diet, including healthier options, and more chances for exercise[56, 57]. However, studies conducted in Brazil highlight the opposite shift in women, with low-income groups transitioning from excessive under-nutrition to obesity within 20 years[50]. Public health messages on obesity prevention and physical activity should target both higher SES adults as well as lower SES adults in low-income countries. This strategy will potentially prevent observed patterns from developed countries where, as high SES adults became more aware of the negative health impact of obesity, consumption of food with low dietary quality was reduced, but similar reductions were not observed among low SES adults, likely due to the greater convenience and lower cost of fast food options compared with higher quality diets[58, 59].

### Strengths and limitations

Our systematic review and meta-analysis identified the lack of consensus among studies focusing on life course SES. The meta-analysis was strengthened by the inclusion of a large number of high-quality studies and participants. Some limitations of the studies included in this systematic review should also be noted. First, life course SES was frequently ascertained through self-reports, especially in cross-sectional studies, leading to potential recall bias. Nevertheless, the results were mostly consistent across cross-sectional and longitudinal studies. Second, given that all the included studies were conducted in developed and upper-middle income countries, the heterogeneity in dietary patterns across regions and countries may portend potentially limited generalizability of these findings. Third, differences in the definition of SES across included studies, with some studies using parental income and education, and others using material wealth, could be a source of heterogeneity in the observed associations. Finally, included studies differed in the type and number of confounders adjusted for in the analysis, raising the possibility of other unmeasured confounders of the associations. Nevertheless, these limitations are unlikely to significantly alter our conclusion since most of the included studies showed consistent associations.

## Conclusion

At least 50% of NCD related deaths are preventable through prevention strategies focused on modifiable risk factors such as obesity, physical activity and nutrition [60–63]. Specifically, recent studies estimate that when started in early life, a large proportion of chronic diseases like cancer and cardiovascular diseases are preventable by reducing obesity and excess weight, and increasing physical activity [62, 63]. This systematic review and meta-analysis summarizes the existing literature on the association between SES and obesity, highlighting the need for public health strategies in population subgroups most vulnerable to obesity (due to easy access to cheap, unhealthy foods and lack of physical activity) and the need for effective strategies that have the best chance of reducing obesity at early ages. The lack of information regarding the association between childhood and life course SES and obesity in developing countries highlights the need for empirical studies to inform obesity prevention strategies in developing countries. **Ethical Approval**: This study was conducted using data from published studies and was exempt from ethical review.

## Supporting information

S1 Fig PRISMA checklist. (DOC)

## Abbreviations

BMI: Body Mass index
HDI: Human Development Index
HIV/AIDS: Human Immunodeficiency Virus/ Acquired Immune Deficiency Syndrome
LMIC: Lower- Middle Income Countries
NCD: Non Communicable Diseases
OR: Odds Ratio
SES: Socio-economic Status
WC: Waist Circumference
WCRF: World Cancer Research Fund
